# Development of an RNA virus-based episomal vector with artificial aptazyme for gene silencing

**DOI:** 10.1007/s00253-024-13327-8

**Published:** 2024-10-18

**Authors:** Ryo Komorizono, Shima Yoshizumi, Keizo Tomonaga

**Affiliations:** 1 Laboratory of RNA Viruses, Department of Virus Research, Institute for Life and Medical Sciences (LiMe), Kyoto University, 53 Kawahara-Cho, Shogo-in, Sakyo, Kyoto, 606-8507 Japan; 2 Laboratory of RNA Viruses, Department of Mammalian Regulatory Network, Graduate School of Biostudies, Kyoto University, 53 Kawahara-Cho, Shogo-in, Sakyo, Kyoto, 606-8507 Japan; 3 Department of Molecular Virology, Graduate School of Medicine, Kyoto University, 53 Kawahara-Cho, Shogo-in, Sakyo, Kyoto, 606-8507 Japan

**Keywords:** Borna virus vector, Viral vector, Aptazyme, Gene regulation

## Abstract

**Abstract:**

RNA virus-based episomal vector (REVec), engineered from Borna disease virus, is an innovative gene delivery tool that enables sustained gene expression in transduced cells. However, the difficulty in controlling gene expression and eliminating vectors has limited the practical use of REVec. In this study, we overcome these shortcomings by inserting artificial aptazymes into the untranslated regions of foreign genes carried in vectors or downstream of the viral phosphoprotein gene, which is essential for vector replication. Non-transmissive REVec carrying GuaM8HDV or the P1-F5 aptazyme showed immediate suppression of gene expression in a guanine or theophylline concentration-dependent manner. Continuous compound administration also markedly reduced the percentage of vector-transduced cells and eventually led to the complete elimination of the vectors from the transduced cells. This new REVec is a safe gene delivery technology that allows fine-tuning of gene expression and could be a useful platform for gene therapy and gene-cell therapy, potentially contributing to the cure of many genetic disorders.

**Key points:**

• *We developed a bornavirus vector capable of silencing transgene expression by insertion of aptazyme*

• *Transgene expression was markedly suppressed in a compound concentration-dependent manner*

• *Artificial aptazyme systems allowed complete elimination of the vector from transduced cells*

## Introduction

Effective therapeutic strategies for overcoming congenital genetic disorders heavily rely on the advancement of gene delivery technology. Compared to immunosuppression methods using low-molecular-weight compounds or enzyme replacement therapy, gene delivery systems are the most realistic and beneficial approach for curing diseases (Bulaklak and Gersbach 2020). Various gene delivery systems have been developed, including cationic polymers or lipid nanoparticles that transduce DNA plasmids or mRNAs (Tsuchida et al. 2024; Wang et al. 2023). Among them, viral vector technologies such as adeno-associated virus (AAV) or lentiviral vectors have contributed substantially to the development of numerous gene or gene-cell therapies (Bulcha et al. 2021; Ehrhardt et al. 2008; Milone and O’Doherty 2018). Viral vector technology has achieved outstanding success in the field of gene delivery systems owing to its high transduction efficiency. However, each type of viral vector has strengths and weaknesses, which can limit its application. Despite rapid technological development, many issues remain, such as cytotoxicity, immune responses, genome contamination of vector sequences, persistence of transgene expression, and transduction efficiency into stem cells (Schambach et al. 2013; Thomas et al. 2003). Thus, there is a need to discover and improve novel viral vectors that can address the above shortcomings.

In previous research, we developed an RNA virus-based episomal vector (REVec) derived from Borna disease virus 1 (BoDV-1) (Daito et al. 2011). BoDV-1 is a nonsegmented negative-strand RNA virus that possesses unique characteristics compared to other RNA viruses (Cubitt et al. 1994). BoDV-1 can establish persistent infections without cell cytotoxicity, allowing for the long-term expression of viral genes (Tomonaga et al. 2002). BoDV-1 replicates in the host nucleus and tethers viral ribonucleoprotein (RNP) complexes to host chromosomes so that viral RNPs are distributed to daughter cells during cell division and can maintain persistent infection (Matsumoto et al. 2012). REVec, which was developed by artificially modifying BoDV-1, achieved sustained replication after transduction into cells and prolonged gene expression in cultured cells and rodent brains (Daito et al. 2011; Komatsu et al. 2020). Compared to other viral vectors, REVec exhibits exceptionally high transduction efficiency in human pluripotent stem cells without inhibiting their cell differentiation ability (Ikeda et al. 2016; Komatsu et al. 2019). Although REVec replicates its RNA within the nucleus, genomic contamination does not occur, unlike in the case of retroviral or lentiviral vectors (Schambach et al. 2013; Vargas et al. 2016). We have already developed a latent REVec that does not produce progeny particles and has generated a safer self-replicating RNA virus vector (Fujino et al. 2017). The characterization of each viral vector is shown in Table 1. This REVec represents a cutting-edge viral vector that overcomes the limitations of existing technologies regarding genomic contamination, persistence of gene expression, and transduction efficiency in stem cells (Fujino et al. 2017; Ikeda et al. 2016).

**Table 1:** Characterization of viral vectors

Vector	Form	Genome contamination	Persistence	Application	Reference
REVec (Bornavirus vector)	RNA	No	Long term	In vivo/ex vivo	(Komatsu and Tomonaga 2020)
Adenovirus vector	DNA	slight	Short term	In vivo	(Scarsella et al. 2024)
Adeno-associated virus vector	DNA	slight	Short term	In vivo	(Zhou et al. 2024)
Retrovirus vector	RNA/DNA	Yes	Long term	In vivo/ex vivo	(Vargas et al. 2016)
Lentivirus vector	RNA/DNA	Yes	Long term	In vivo/ex vivo	(Arsenijevic et al. 2022)
Sendai virus vector	RNA	No	Short term	In vivo	(Nakanishi and Otsu 2012)

On the other hand, the system that allows REVec to control gene expression or vector elimination is not well established. After REVec is transduced into cells, the vector RNA automatically replicates in the nucleus and continuously initiates transcription, making it difficult to control gene expression and eliminate the vector. A novel REVec that can switch off gene expression could be a safer and more broadly applicable gene delivery technology. Ideally, such a system would allow the fine-tuning of expression levels according to individual patient needs and provide a safety switch that would reduce the risk of undesirable immune reactions to therapeutic transgenes.

Previously, we reported a system for the regulation of gene expression in REVec using the L2bulge9 ribozyme (Win and Smolke 2007; Yamamoto et al. 2019). This REVec-L2bulge9 system is a switch-on vector that enables an increase in gene expression levels by inhibiting RNA self-cleavage of target genes via the administration of theophylline. Although this system has the advantage of regulating gene expression, it is not practical for gene therapy because continuous administration of ligands is necessary to induce the expression of the target gene. In addition, since the L2bulge ribozyme acts only on the target gene, the vector remains in transduced cells regardless of the presence or absence of theophylline (Win and Smolke 2007). To further increase the safety of REVec, it is desirable to develop a novel system that can switch off gene expression.

Artificial aptazymes, which integrate aptamers that bind to small-molecule compounds and ribozymes that induce self-cleavage, are attracting attention as RNA devices that facilitate the control of target gene expression (Link and Breaker 2009; Zhang et al. 2010). In addition to the additional expression of promoter-regulator proteins or the addition of destabilizing domains, aptazymes are attractive because they are independent expression regulators that act directly on mRNAs (Wieland and Hartig 2008a). In many cases, the aptazyme is inserted into the untranslated region of the target gene, and compound administration triggers self-cleavage that leads to the silencing of expression (Chang et al. 2012; Soukup and Breaker 1999). This switch is immediate, and gene expression can be flexibly controlled. Recent progress in screening methods has led to the development of new highly efficient aptazymes, and synthesized aptazymes with increased specificity and expression suppression ability, such as the GuaM8HDV and P1-F5 aptazymes, have been identified (Auslander et al. 2010; Nomura et al. 2013). The triggers of these aptazymes, guanine, and theophylline have high cell permeability and low cytotoxicity, making them highly convenient and easy to use in vivo (Lanznaster et al. 2016; Tsai and Liu 2004). Thus, these artificial aptazymes can be expected to enable the control of gene expression induced by viral vectors, including REVec.

In this study, two highly efficient aptazymes, GuaM8HDV and P1-F5, were applied to develop a novel REVec capable of controlling transgene expression. GuaM8HDV is an efficient guanine-activated aptazyme that exhibits a high ON/OFF ratio, designed by inserting a guanine aptamer into the P4 stem region of the ribozyme derived from the hepatitis delta virus (HDV) (Nomura et al. 2013). P1-F5 is an artificial theophylline-activated aptazyme based on the hammerhead ribozyme (Auslander et al. 2010). We focused on in vitro experiments to demonstrate the potential of these aptazymes to suppress gene expression and eliminate vectors from the transduced cells. The expression of the transgene was markedly suppressed in a compound concentration-dependent manner, and an immediate inhibition of expression was achieved. Notably, the insertion of an aptazyme downstream of the viral phosphoprotein (P) gene resulted in the complete elimination of the vector from the transduced cells, with vector antigens and RNAs below detection limits. We have achieved fine-tuning of gene expression in vectors with artificial aptazymes, providing a more secure REVec.

## Materials and methods

### Cell culture and reagents

Vero (ATCC CCL-91) cell lines were maintained in Dulbecco’s modified Eagle’s medium (DMEM; Thermo Fisher Scientific) supplemented with 2% heat-inactivated fetal calf serum (FCS; MP Biomedical) and 100 U/mL penicillin–streptomycin (Nacalai Tesque). The HEK293T (ATCC CRL-11268) and A549 (ATCC CCL-185) cell line was maintained in DMEM supplemented with 10% heat-inactivated FCS, 100 U/mL penicillin–streptomycin, and 100 mM nonessential amino acids (Thermo Fisher Scientific). The cells were maintained at 37 °C in a humidified incubator containing 5% CO_2_. Guanine (Tokyo Chemical Industry) and theophylline (Tokyo Chemical Industry) were dissolved in 0.2 M NaOH (Nacalai Tesque) and dimethyl sulfoxide (DMSO), respectively, for the preparation of the stock solution.

### Production and preparation of the REVec

Recombinant transmission-defective Borna disease virus vectors lacking both matrix (M) and glycoprotein (G) genes (REVec ΔMG) were obtained by a reverse genetics system as reported previously (Fujino et al. 2017; Kanda et al. 2022). These vectors encoded eGFP or NanoLuc (Promega) as a reporter gene between the P and L genes with an additional transcription cassette. Briefly, HEK293T cells were transfected with BoDV-1 cDNA-expressing vector plasmids and helper plasmids expressing the BoDV-1 N, P, L, M, and G genes. At 4 days post-transfection, cell-free vector solutions were prepared by sonication as previously described (Fujino et al. 2017). Briefly, REVec-transduced cells were suspended in OptiMEM (Invitrogen) and subjected to sonication using a BIORUPTOR II (Sonic Bio). After centrifugation of the sonicated cell suspensions at 1200 × *g* for 25 min at 4 °C, the supernatant was collected and stored at − 80 °C as a cell-free vector solution.

### Plasmid construction

To prepare the luciferase gene expression plasmids with the insertion of aptazyme sequences, the sequence of GuaM8HDV or P1-F5 aptazyme was added to the N- (+ N) or C terminus (+ C) or both ends (+ NC) of the NanoLuc gene (Promega) by PCR extensions and cloned into pcDNA3 (Invitrogen), respectively (Auslander et al. 2010; Nomura et al. 2013). The PEST sequences were added to these NanoLuc genes at the C terminus to shorten the half-life of the protein and to analyze the variation in gene expression with high sensitivity (Rogers et al. 1986). To prepare REVec ΔMG plasmids encoding the GFP gene or luciferase gene with aptazymes, each gene was inserted into *Bst*BI and *Pac*I site between the P and L genes in REVec plasmids (Fujino et al. 2017). The sequences of the aptazymes used were as follows: GuaM8HDV aptazyme (Nomura et al. 2013): 5’-ATGGCCGGCATGGTCCCAGCCTCCTCGCTGGCGCCGGCTGGGCAATGCTATAATCGCGTGGATATGGCACGCAAGTTTCTACCGGGCACCGTAAATGTCCGACTAGTAGCGAATGGGACGCACAAATCTCTCTAG-3’; P1-F5 aptazyme (Auslander et al. 2010): 5’-CTGAGGTGCAGGTACATCCAGCTGACGAGTCCCAAATAGGACGAAAGCC ATACCAGCCGAAAGGCCCTTGGCAGGGTTCCTGGATTCCACTGCTATCCAC-3’.

### Western blotting

Cell lysates for Western blotting analysis were prepared as previously described (Komorizono et al. 2020). Cultured cells were lyzed with SDS sample buffer. Cell lysates were subjected to SDS–PAGE on Any kD Mini-PROTEAN TGX Precast Protein Gels (Bio-Rad) after sonication using a BIORUPTOR II (Sonic Bio). Proteins in the SDS–PAGE gels were transferred to a Trans-Blot Turbo polyvinylidene difluoride transfer pack (Bio-Rad). The transfer membranes were blocked with Blocking One (Nacalai Tesque) and then reacted with anti-BoDV-1 N (rabbit polyclonal HB01), anti-BoDV-1 P (rabbit polyclonal HB03) (Watanabe et al. 2000), and anti-Tubulin (B-5–1-2; Sigma–Aldrich) antibodies diluted with Can Get Signal Immunoreaction Enhancer Solution (Toyobo) at room temperature for 1 h. After washing with TBS-T (Tris-buffered saline, 0.1% Tween 20) buffer for 1 h, the membranes were incubated with horseradish peroxidase-conjugated secondary antibodies (Jackson ImmunoResearch) at room temperature for 1 h. The signals of the chemiluminescence reaction were detected using ECL Plus Western blotting detection reagents (GE Healthcare) and a Fusion Solo instrument (Vilber-Lourmat). The images of the captured membrane were analyzed using ImageJ for quantification of band intensities.

### qRT–PCR analysis

Total RNA was extracted using an RNeasy Mini Kit with DNase I treatment (Qiagen) according to the manufacturer’s instructions. Reverse transcription was performed using a Verso cDNA synthesis kit (Thermo Fisher Scientific) with oligo dT primers for vector mRNA or vector genome-specific primers for vector genomic RNA. The qRT–PCR analyses were performed using Luna Universal qPCR Master Mix (New England Biolabs). The sequences of the primer pairs used were as follows: REVec forwards: 5’-ATGCATTGACCCAACCGGTA-3’, reverse: 5’-ATCATTCGATAGCTGCTCCCTTC-3’; NanoLuc forwards: 5’-GTCCTGAGCGGTGAAAATGG-3’, reverse: 5’-CGTAACCCCGTCGATTACCA-3’; GAPDH forwards: 5’-ATTTGGCTACAGCAACAGGGT-3’, reverse: 5’-AACTGTGAGGGGAGATTCAGTG-3’; and REVec genomic RNA-specific RT primer: 5’-TGTTGCGTTAACAACAAACCAATCAT-3’.

### Immunofluorescence analysis

Cells were fixed with 4% paraformaldehyde solution (Nacalai Tesque) for 1 h after removal of the culture medium and permeabilized by incubation in PBS containing 0.25% Triton X-100 for 10 min. After cell permeabilization, the fixed cells were incubated with anti-BoDV-1 N (rabbit polyclonal HB01) or anti-BoDV-1 P (rabbit polyclonal HB03) antibodies at room temperature for 90 min (Watanabe et al. 2000). This incubation step was followed by incubation with the appropriate Alexa Fluor-conjugated secondary antibodies (Thermo Fisher Scientific) and DAPI (4’,6-diamidino-2-phenylindole; Merck) at room temperature for 1 h after washing with PBS. An ECLIPSE Ti confocal laser scanning microscope (Nikon) was used for imaging and data collection.

### WST-1 assay

To evaluate the cytotoxicity of compounds and to measure the cell viability, WST-1 assay was performed in 96 well plates after seeding culture cells. WST-1 activity was measured using the GloMax Discover System (Promega) and a Premix WST-1 Cell Proliferation Assay System (TaKaRa) according to the manufacturer’s instructions.

### Luciferase assay

To quantify the expression level of NanoLuc encoded in viral vectors, NanoLuc activity was measured with a Nano-Glo Luciferase Assay System (Promega) and normalized to the corresponding WST-1 activity for measuring cell viability.

### Statistical analysis

All of the statistical analyses and calculations of the significance values were performed using GraphPad Prism 10 (GraphPad). The statistical tests used in each experiment are shown in the figure legends.

## Results

### Verification of the ability of artificial aptazymes to regulate gene expression

**Fig. 1:**
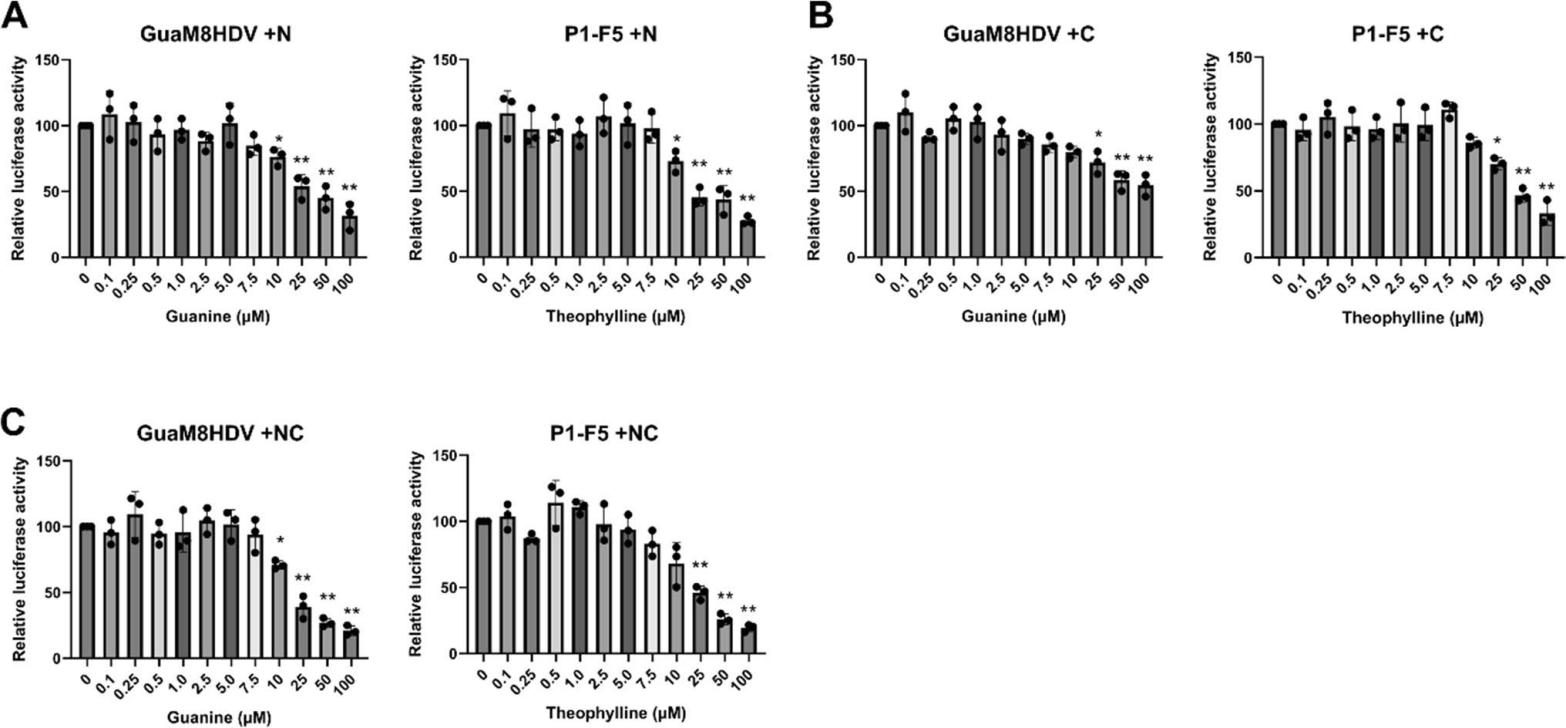
Regulation of gene expression by insertion of aptazymes into plasmids. **A** Relative luciferase activity after the addition of compounds that react with aptazymes in plasmid-transfected cells. After HEK293T cells were transfected with 10 ng of plasmids encoding NanoLuc with GuaM8HDV or the P1-F5 aptazyme, guanine or theophylline was added at each concentration, and luciferase activity was measured 4 days later. The aptazyme was inserted into the N terminus (+ N) of the NanoLuc gene. **B** Relative luciferase activity for plasmids in which the aptazyme was inserted at the C terminus (+ C) of the NanoLuc gene. **C** Relative luciferase activity for plasmids in which the aptazyme was inserted at both ends (+ NC) of the NanoLuc gene. The values are expressed as the means ± SEs of the results from three biologically independent replicates. Significance was analyzed by Dunnett’s multiple-comparison test. *, *p* < 0.01, **, *p* < 0.001

To investigate the regulation of gene expression by aptazyme, we first measured the efficacy of expression reduction using luciferase expression plasmids. In this study, we employed two types of highly efficient aptazyme. The GuaM8HDV or P1-F5 aptazyme was inserted into the terminus of the gene of interest. RNA self-cleavage of aptazymes is induced by treatment with low-molecular-weight compounds, resulting in immediate suppression of gene expression (Auslander et al. 2010; Nomura et al. 2013). The GuaM8HDV and P1-F5 aptazymes are activated by the addition of guanine and theophylline, respectively. To enable a more efficient inhibition of gene expression, we investigated the insertion sites of the aptazymes. After transfecting HEK293T cells with plasmids encoding the GuaM8HDV or P1-F5 aptazyme-inserted upstream, downstream, or at both ends of the luciferase gene, luciferase activity was measured (Fig. 1). After the administration of the compounds at various concentrations, we observed a concentration-dependent decrease in the activity in all groups where the aptazyme was inserted upstream (+ N group, Fig. 1A), downstream (+ C group, Fig. 1B), or at both ends (+ NC group, Fig. 1C). However, the + NC groups with aptazymes inserted at both ends showed a significantly greater decrease in activity at the same concentration than the + N and + C groups (Fig. 1C). These results suggest that the insertion of aptazymes at both ends of the gene of interest can more efficiently control gene expression in viral vectors.

### Development of a REVec that enables the suppression of transgene expression

**Fig. 2:**
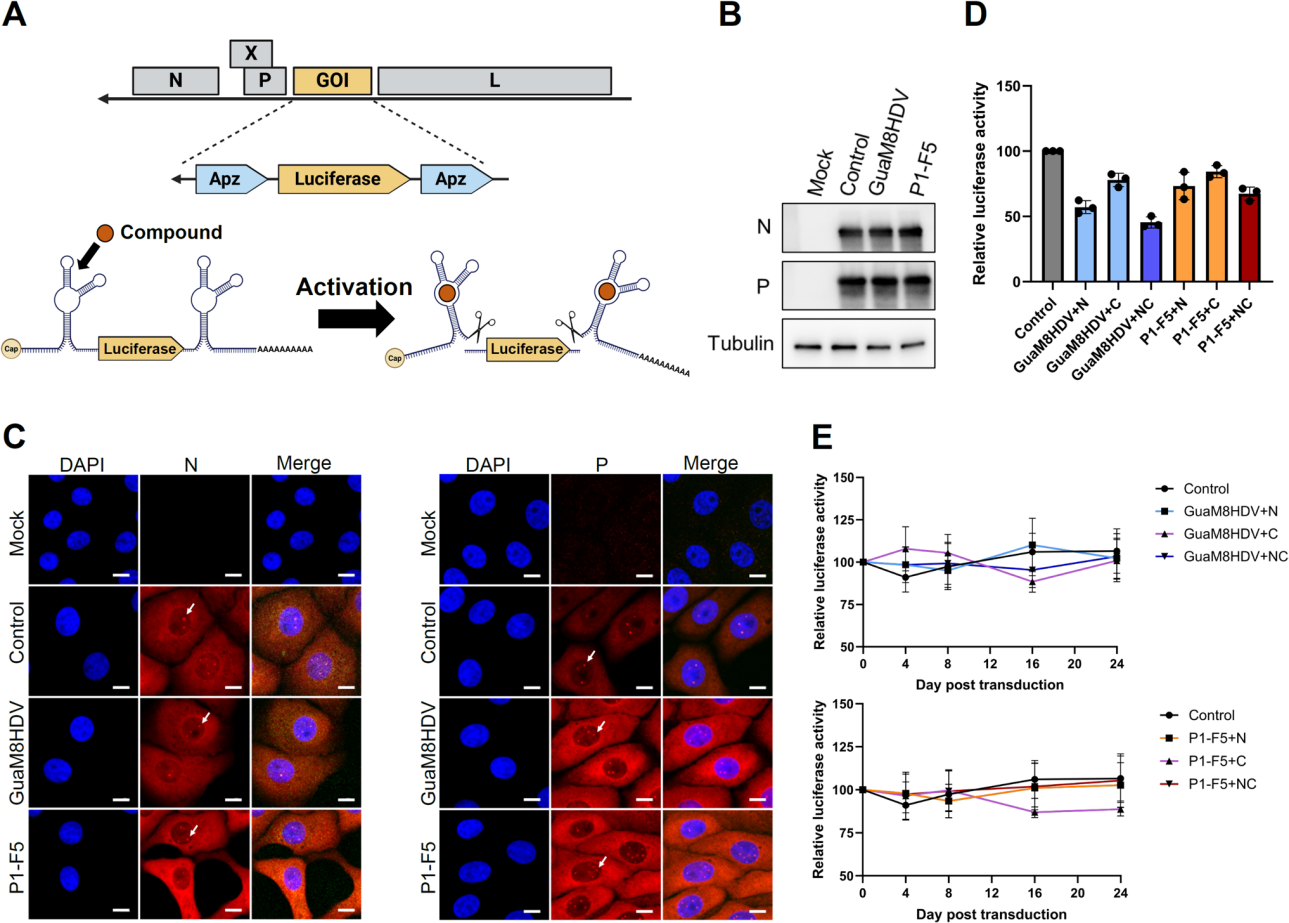
Construction of aptazyme-containing REVec with controllable gene expression. **A** Schematic showing that aptazymes enable the suppression of gene expression induced by REVec. Administration of the aptazyme ligand induces self-cleavage of the untranslated regions at both ends of the luciferase gene, resulting in the shutdown of gene expression. **B** Detection of antigens in REVec-transduced cells. After the transduction of REVec with Vero cells at an MOI of 0.25, the N and P proteins were detected by Western blot analysis. The results are shown for REVec-transduced cells with GuaM8HDV or P1-F5 aptazymes inserted at both ends of the NanoLuc gene. **C** Subcellular localization of the N and P proteins of the REVec in transduced cells. REVec with aptazymes added to both ends of the luciferase gene was transduced into Vero cells at an MOI of 0.25, and the subcellular localization of the N and P proteins was analyzed by IFA. The white arrows point to vSPOT in transduced cells. Scale bar, 10 μm. **D** Relative luciferase activity in cells transduced with the aptazyme-containing REVec. Each vector was transduced into Vero cells at an MOI of 0.25, after which luciferase activity was measured 3 days later. + N, + C, and + NC indicate the insertion sites of the aptazymes. **E** Evaluation of the effect of aptazyme insertion on the persistence of gene expression. Each vector was transduced into Vero cells at an MOI of 0.25, and luciferase activity was subsequently measured at each time point. The values are expressed as the means ± SEs of the results from three biologically independent replicates

To develop a novel REVec that allows for the control of transgene expression levels, the GuaM8HDV and/or P1-F5 aptazymes were inserted at both ends (+ NC) or either end (+ N and + C) of the luciferase gene (Fig. 2A). The transgene with aptazymes was encoded between the viral P and L genes of the vector. This REVec is a non-transmissive vector that does not produce progeny vector particles because it lacks the M and G genes. REVec was produced using a reverse genetics system and transduced into Vero cells (Fig. 2B) (Fujino et al. 2017). In these transduced Vero cells, a nuclear structure with N and P proteins as structural components, called vSPOT, was observed (Fig. 2C, arrows) (Hirai et al. 2016, 2021; Matsumoto et al. 2012). This vSPOT formation suggested that the vector was successfully transcribed and replicated in the transduced cells (Hirai et al. 2016). On the other hand, the insertion of aptazymes reduced luciferase activity in REVec-transduced cells by approximately one-half to one-third compared to that in control cells (Fig. 2D). Furthermore, no attenuation of gene expression was observed for at least 24 days after REVec transduction (Fig. 2E). These results indicate that the insertion of aptazymes does not inhibit persistent gene transduction by REVec.

### Regulation of gene expression by aptazymes in REVec-transduced cells

**Fig. 3:**
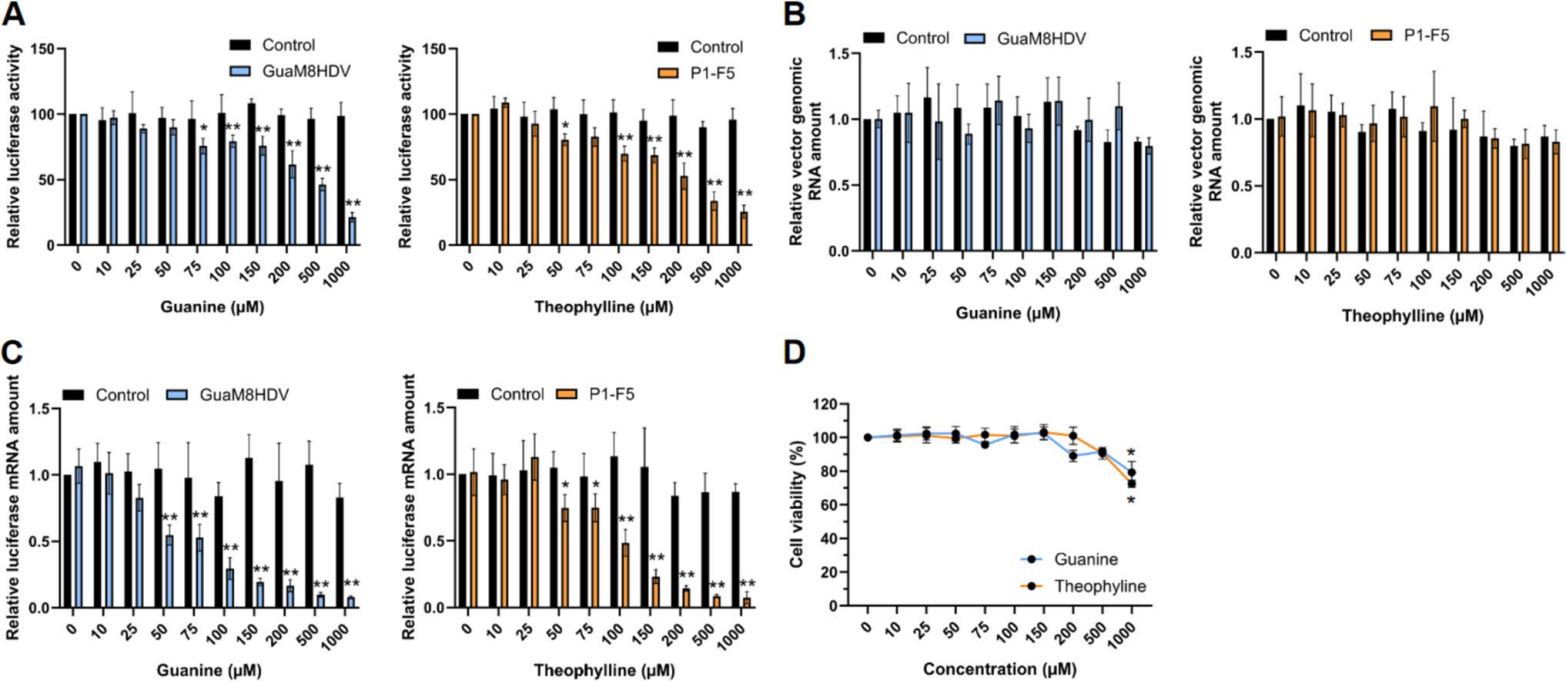
Aptazymes enable the suppression of gene expression induced by REVec. **A** Aptazyme enable the suppression of gene expression induced by REVec. After transduction into Vero cells with aptazyme-inserted REVec (+ NC) at an MOI of 0.25, the transduced cells were treated with guanine or theophylline at various concentrations. Relative luciferase activity was measured at 4 days post-administration. After treatment of transduced cells with the compounds, the amount of genomic RNA in the REVec (**B**) and the amount of mRNA in the luciferase gene (**C**) were quantified by qRT–PCR. Each value was normalized to the Ct value of the GAPDH gene. **D** Cytotoxicity of guanine and theophylline. Cell viability was measured at 4 days post-administration. The data are presented as the means + SEs of results from three independent experiments. Significance was analyzed by Dunnett’s multiple-comparison test. *, *p* < 0.01, **, *p* < 0.001

Next, the potential to suppress gene expression by compound treatment was examined in cells transduced with the aptazyme-containing REVec generated as described above. After the transduction of REVec encoding aptazymes added to both ends of the luciferase gene (+ NC) into Vero cells, the reduction in luciferase activity caused by treatment with guanine or theophylline was quantified. As shown in Fig. 3A, luciferase activity decreased significantly in a concentration-dependent manner, with activity decreasing to less than approximately 25% of that of the control without aptazyme insertion after 4 days of administration. To determine whether RNA cleavage by the aptazyme was specifically induced in the luciferase mRNA after administration, the amounts of vector genomic RNA and luciferase mRNA were measured in the presence or absence of the compounds (Fig. 3B and C). No significant difference was observed in the vector genome RNA levels upon compound administration (Fig. 3B), whereas the luciferase mRNA levels significantly decreased in a concentration-dependent manner (Fig. 3C). No changes in luciferase mRNA levels were observed in the control without aptazyme insertion. Moreover, the relatively low cytotoxicity of the compounds suggested that the decrease in vector RNA was not due to cell death, but rather to the suppression of gene expression by aptazyme cleavage. These results indicate that transgene expression from REVec can be inhibited through mRNA cleavage by activating the GuaM8HDV and P1-F5 aptazymes via the administration of guanine and theophylline, respectively.

### Development of REVec that enables vector elimination from transduced cells

The above results suggested that aptazymes are useful for the immediate control of gene expression induced by REVec. This strategy is expected to be applicable not only for the control of transgene expression but also for vector elimination from transduced cells. Since the expression of the N, P, and L genes is essential for the replication of REVec, suppressing the expression of any of these genes could reduce the replication efficiency of REVec and potentially achieve complete elimination of the vector from the transduced cells (Schwemmle et al. 1997; Whelan et al. 2004). Therefore, a novel REVec with an aptazyme-inserted downstream of the P gene was developed to examine whether vector elimination from transduced cells could be achieved by inducing the cleavage of P gene mRNA.

**Fig. 4:**
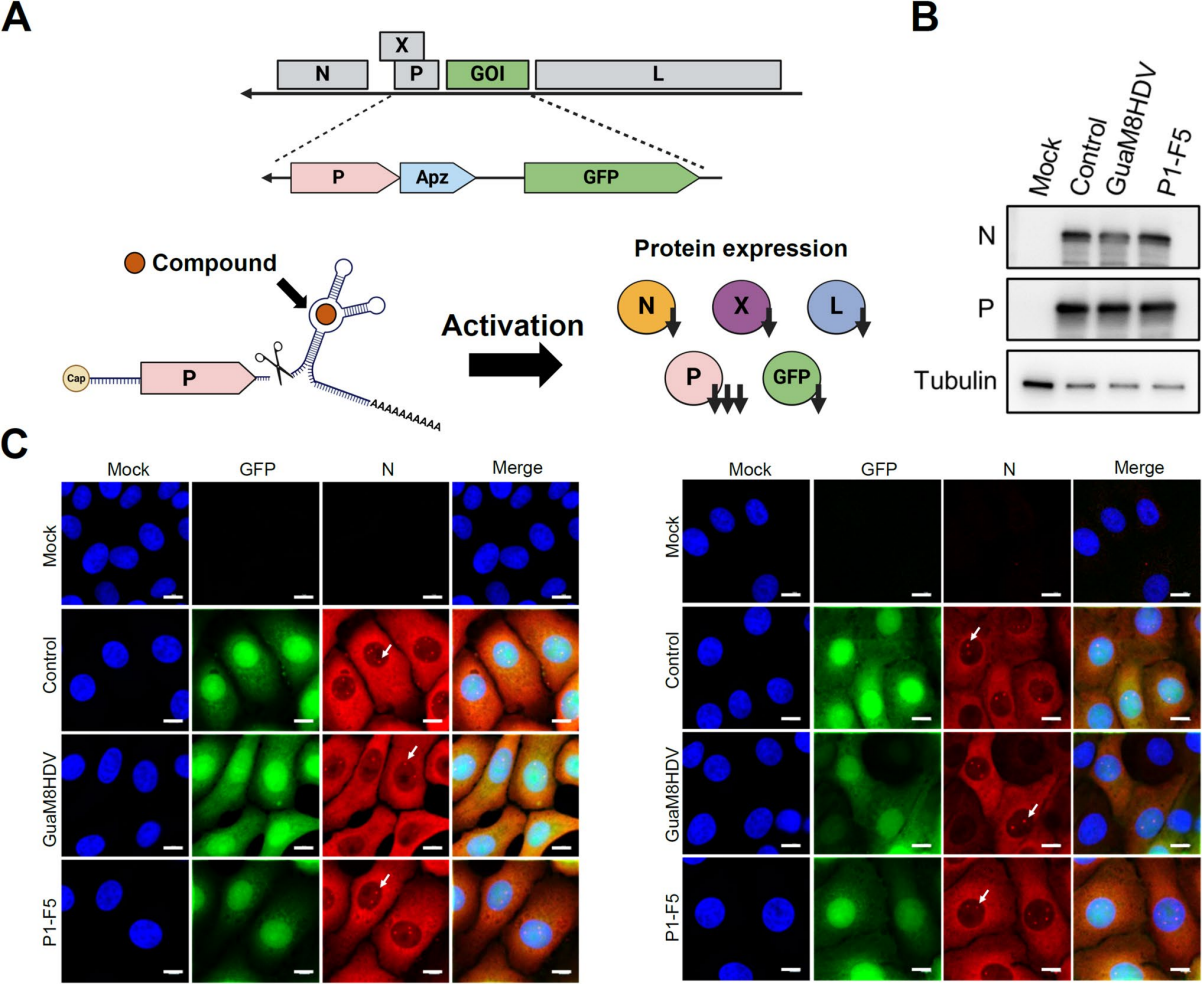
Aptazymes can eliminate REVec from transduced cells. **A** Schematic showing that aptazymes can eliminate REVec from transduced cells. The GuaM8HDV or P1-F5 aptazyme was inserted downstream of the P gene of REVec and induced self-cleavage of the mRNA after aptazyme activation via the administration of each compound. **B** Detection of antigens in REVec-transduced cells. After the transduction of REVec with Vero cells at an MOI of 0.25, the N and P proteins were detected by Western blot analysis. **C** Subcellular localization of the N and P proteins of the REVec in transduced cells. Each vector with aptazymes inserted downstream of the P gene was transduced into Vero cells at an MOI of 0.25, and the subcellular localization of the N and P proteins was analyzed by IFA. The white arrows point to vSPOT in transduced cells. Bar, 10 μm

**Fig. 5:**
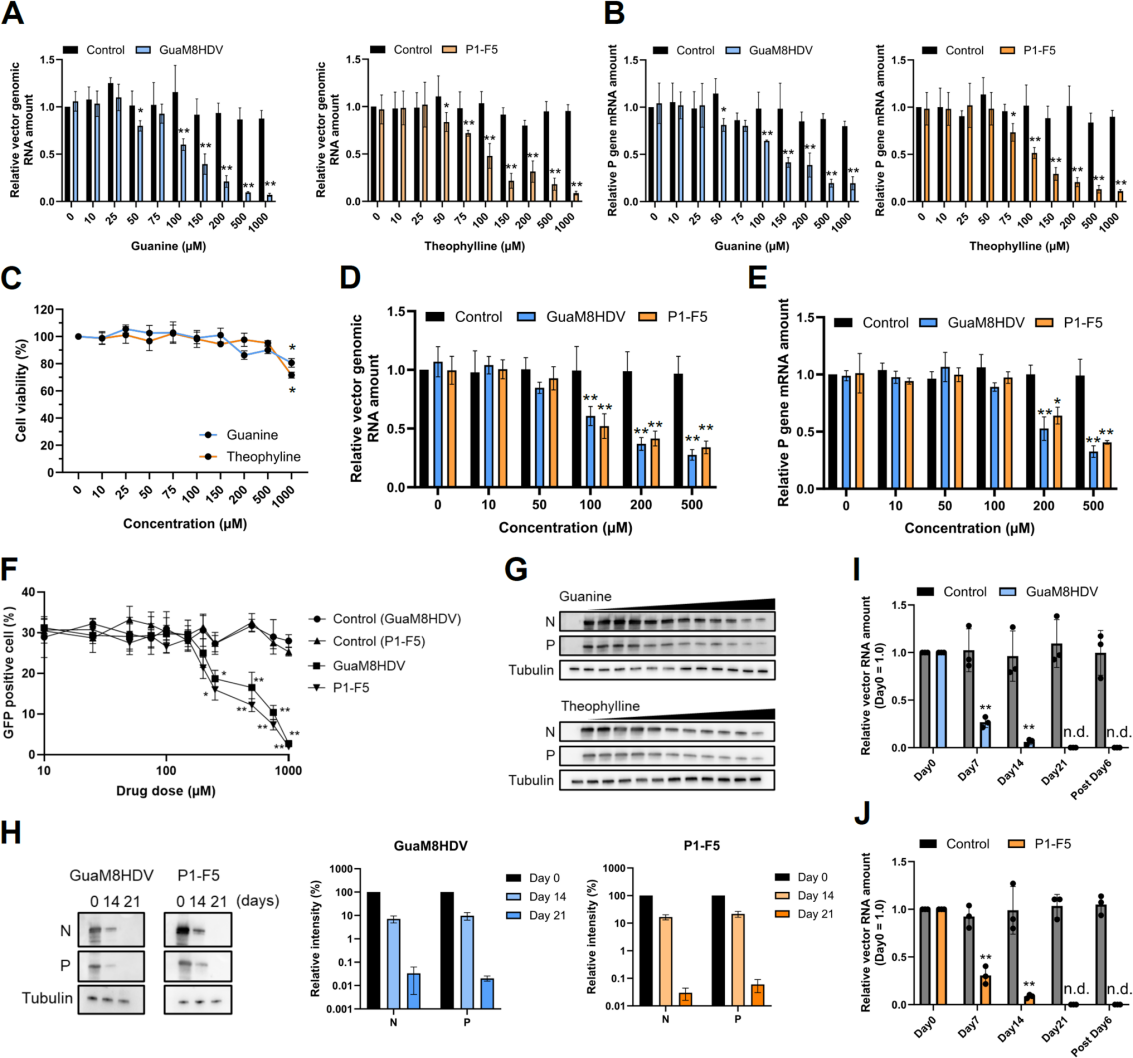
Aptazyme enables the complete elimination of REVec from transduced cells. **A** Suppression of P gene expression by aptazymes leads to elimination of the genomic vector RNA. After transduction into Vero cells with aptazyme-inserted REVec at an MOI of 0.25, the transduced cells were treated with guanine or theophylline at various concentrations. The amount of genomic vector RNA of the REVec (**A**) and the mRNA of the P gene (**B**) were quantified by qRT–PCR at 4 days post-administration. Each value was normalized to the Ct value of the GAPDH gene. **C** Cytotoxicity of guanine and theophylline. Cell viability of A549 cells was measured at 4 days post-administration. After transduction into A549 cells with aptazyme-inserted REVec at an MOI of 0.2. The amount of genomic vector RNA of the REVec (**D**) and the mRNA of the P gene (**E**) were quantified by qRT–PCR at 4 days post-administration. Each value was normalized to the Ct value of the GAPDH gene. **F** Dose-dependent decrease in the percentage of vector-transduced cells. HEK293T cells were transduced with each vector at an MOI of 0.3. At 8 days post-administration, the percentage of GFP-positive cells was measured by a Tali cytometer and IFA. **G** Decrease in the amount of vector antigen caused by aptazyme activation. After compound administration at each concentration and aptazyme activation, N and P proteins were detected by WB at 4 days post-administration. The right lane indicates higher compound concentrations. **H** The amounts of N and P protein at 0, 14, and 21 days post-administration. After the administration of guanine or theophylline at 200 μM to transduced cells, the cells were passaged every 3 days. N and P proteins in transduced Vero cells were detected by Western blot analysis. The right graphs show the quantification and comparison of band intensities. The data were normalized to the band intensity of tubulin. The analysis was performed by ImageJ. (I) Relative amount of genomic vector RNA of GuaM8HDV vector and (J) P1-F5 vector after continuous compound administration. After the continued administration of guanine or theophylline at 200 μM to transduced cells for 0, 7, 14, or 21 days, the amount of the genomic vector RNA was quantified via qRT–PCR. After continuing treatment with the compound for 21 days, the cells were cultured in the absence of the compound for 6 days, after which the amount of genomic RNA was quantified in the same manner. Each value was normalized to the Ct value of the GAPDH gene. The data are presented as the means and + SEs of results from three independent experiments. Significance was analyzed by Dunnett’s multiple-comparison test. *,* p* < 0.01, **, *p* < 0.001. n.d., not detected

We generated REVec encoding GFP as a marker with the GuaM8HDV or P1-F5 aptazyme-inserted downstream of the P gene (Fig. 4A and B). As shown in Fig. 2, intranuclear structures, vSPOT, in which N and P proteins localize, were observed in transduced cells (Fig. 4C). Therefore, after transducing REVec into Vero cells, the changes in the amount of vector genomic RNA and P gene mRNA were verified after the administration of the compounds (Fig. 5A and B). As expected, both the amount of vector genomic RNA (Fig. 5A) and P gene mRNA (Fig. 5B) decreased significantly in a concentration-dependent manner and decreased to a maximum of approximately 15% or less. To validate the cell type dependency, we examined the suppression of gene expression by aptazymes using lung-derived A549 cells. Similar to previous results, a marked decrease in vector RNA was observed with low cytotoxicity (Fig. 5C, D, E). These results suggested that the mRNA level of the P gene decreased due to RNA cleavage by aptazyme activation, and the vector genome RNA decreased due to reduced vector replication efficiency caused by the lack of P gene expression.

Next, we verified the reduction in the percentage of REVec-transduced cells due to compound administration (Fig. 5F). Using GFP fluorescence as a marker, the percentage of REVec-transduced cells at each concentration was quantified by counting the number of positive cells by immunostaining. Following the administration of compounds at various concentrations with a starting point of 30% positivity, the percentage of GFP-positive cells was significantly lower in the vector with aptazyme group than in the control group. This finding suggested that vector replication was inhibited by RNA cleavage due to aptazyme activation, as mentioned above. Furthermore, the amounts of the vector antigen and the N and P proteins decreased in a concentration-dependent manner (Fig. 5G). After administering the compound to REVec-transduced cells for 21 days, the level of the vector antigen was below the detection limit according to Western blot analysis on day 21 (Fig. 5H). Similarly, the amount of vector genomic RNA and mRNA was below the detection limit according to qRT–PCR analysis (Fig. 5I and J). Notably, after administering the compound for 21 days and then culturing the transduced cells for 6 days without the compound, no vector RNA was detected. After these treatments, the episomal state of REVec was not maintained and the vector was cleared from the cells. These results suggested that vector elimination from transduced cells is possible through the suppression of vector gene expression using aptazymes. REVec, which is capable of expression control and elimination by aptazymes, is expected to be a safer gene transfer system.

## Discussion

Since the control of gene expression from viral vectors is a critical issue that directly affects the safety of gene therapy drugs, many systems for controlling gene expression have been developed, particularly lentiviral and AAV vectors (Matrai et al. 2010; Page et al. 2020; Parr-Brownlie et al. 2015; Tickner and Farzan 2021). They deliver DNA to induce gene expression in transduced cells, so promoter-driven expression control is useful. As representative examples, the Tet-on system using Tet-responsive element (TRE) and tetracycline-regulated transactivator (tTA) fused with TetR and VP16AD and the Tet-off system using reverse tetracyclin-regulated transactivator (rtTA) and TRE have been successfully used for doxycycline-dependent regulation in vitro and in vivo (Urlinger et al. 2000). Similarly, a regulatory system involving the synthetic steroid mifepristone (Mfp) and the chimeric transactivator GLVP has also been applied (Ngan et al. 2002; Wang et al. 1997). This transactivator GLVP is a fusion protein composed of a VP16 transactivation domain, a Gal4 DNA-binding domain (Gal4 DBD), and a human progesterone receptor ligand-binding domain called PRLBD-891. Through the interaction of Mfp with the PRLBD-891 domain, the transactivator forms a dimer, the Gal4 DBD binds to the Gal4 upstream activating sequence (UAS), and mRNA transcription is subsequently induced (Marmorstein et al. 1992; Vegeto et al. 1992; Wang et al. 1997). As described above, gene expression in DNA-mediated viral vectors can be controlled by regulating promoter activity, but these systems require the co-expression of controller proteins such as tTA and GLVP in addition to the target gene.

On the other hand, the aptazyme used in this study has a wide range of applications because it is not a promoter-driven mechanism for gene expression control but a system that acts on RNA (Soukup and Breaker 1999; Zhang et al. 2010). Aptazymes have been employed in several RNA virus-based vectors, such as Vesicular stomatitis virus, and measles virus vectors, as well as lentiviral vectors and AAV vectors, to enable expression control (Ketzer et al. 2014; Takahashi and Yokobayashi 2019). The regulation of gene expression by aptazymes is induced by the administration of low-molecular-weight compounds such as guanine and theophylline and thus is highly feasible for clinical trials (NCT01263106, NCT02184247) in terms of cell permeability and toxicity (Lanznaster et al. 2016; Tsai and Liu 2004). Recent advances in screening methods for aptazyme optimization have led to the identification of artificially designed aptazyme sequences with high specificity and cleavage efficiency (Kobori et al. 2017; Nomura and Yokobayashi 2015; Rehm et al. 2021; Wieland and Hartig 2008b; Zhong et al. 2016). The GuaM8HDV aptazyme is an artificial device modified from the genomic HDV ribozyme, with the P4-L4 stem-loop region fused to the RNA aptamer at the connector stem sequences (Nomura et al. 2013). The substantial improvement in the regulatory dynamic range (ON/OFF ratio) in vitro has led to new possibilities for this aptazyme. The P1-F5 aptazyme is an artificial RNA device designed by fusing the optimized hammerhead ribozyme with a theophylline aptamer (Auslander et al. 2010). The P1-F5 aptazyme has improved theophylline specificity by modifying the codon and the sequence in the stem-loop region. The use of these aptazymes is intended to suppress gene expression by fragmentation of the 5′ cap structure or poly A tail of the target mRNA (Ketzer et al. 2014). In this study, aptazymes also contributed to the control of gene expression and vector elimination by insertion into the untranslated region of target mRNAs. The administration of guanine and theophylline alone did not change vector RNA levels or luciferase activity (the control groups in Fig. 3 and Fig. 5), suggesting that RNA cleavage by aptazyme activation was responsible for vector elimination. However, it has been reported that the inhibition efficacy of gene expression by aptazymes is greatly affected by their insertion position, and aptazymes inserted into the 5′ untranslated region are more efficient at suppressing expression than those inserted into the 3′ untranslated region (Ketzer et al. 2014; Takahashi and Yokobayashi 2019). Trimming of the cap structure has a stronger effect on mRNA destabilization and inhibition of translation initiation than removal of the poly A tail (Tickner and Farzan 2021). Similarly, in this study, Fig. 1 A and B show that the suppression efficacy of gene expression was greater in the group with the aptazyme inserted in the N terminus (+ N) than in the group with the aptazyme inserted in the C terminus (+ C). In addition, as shown in Fig. 2D, optimization of the aptazyme sequence and insertion position is needed in the future because it has a negative effect on the gene expression level of the vector.

There are several reports on the regulation of REVec-induced expression and vector elimination. Inhibition of viral polymerase activity by antiviral drugs and RNA degradation by vector-specific small interfering RNA may help control REVec in vitro. ribavirin and favipiravir have shown antiviral activity against bornaviruses and thus have potential for the control of REVec (Jordan et al. 1999; Mizutani et al. 1998; Tokunaga et al. 2017). However, ribavirin is highly cytotoxic, and favipiravir must be administered at high concentrations for long periods (Jordan et al. 1999; Mizutani et al. 1998). Additionally, unlike the guanine and theophylline used in this study, favipiravir has low permeability into the brain and is expected to have limited efficacy in regulating gene expression in the central nervous system (Rong et al. 2023). In fact, the administration of favipiravir failed to inhibit the replication of the rabies virus in the central nervous system in animal experiments (Banyard et al. 2019; Yamada et al. 2019). In terms of cytotoxicity and biodistribution, the regulation of gene expression by aptazymes has advantages over the use of these antiviral drugs. The application of small interfering RNA (siRNA) is another powerful tool for REVec control. Bornavirus-specific siRNA cocktails in combination with favipiravir suppressed Borna disease virus infection and gene expression in vitro (Teng et al. 2019). However, drug delivery systems are a major barrier to siRNA application, and their practical potential is currently limited (Paunovska et al. 2022). It is also necessary to study the regulation of REVec by microRNAs in the future. Similar to aptazyme strategy, insertion of a complementary sequence to the microRNA into the vector would induce RNA degradation by microRNA expression in the host and achieve the control of gene expression. This microRNA strategy is highly potential for REVec because it has been used successfully with lentiviral vectors and Sendai virus vectors (Brown et al. 2007; Nishimura et al. 2017; Sano et al. 2016). The regulation of REVec-induced gene expression by aptazymes is expected to have a wide range of applications as a safe and practical system. As we previously reported, the L2bulge9-REVec system is a switch-on vector that induces gene expression via the administration of theophylline (Yamamoto et al. 2019). Together with this study, we have successfully established a REVec construct that enables both the induction and suppression of gene expression. These safer systems would dramatically increase the potential of REVec as a novel useful platform for gene therapy and gene-cell therapy.

We have demonstrated that aptazymes can control REVec-induced gene expression, but the concentrations of the applicable compounds are relatively high, and there is room for improvement. Further consideration of the insertion position of aptazymes, such as at the terminus or in the middle of the N or L gene of REVec, may increase the efficiency of expression regulation and vector elimination. The use of improved aptazymes with higher specificity and cleavage efficiency would allow rapid regulation of gene expression and vector elimination at lower concentrations. In addition to aptazymes, chemogenetic ON/OFF switches using proteases or optogenetic tools could be applied to REVec (Heilmann et al. 2021; Tahara et al. 2019). By using these systems, REVec will lead to the development of numerous gene therapy drugs as a platform that can flexibly manipulate gene expression levels.

In this study, we developed a novel REVec that is capable of expression control and vector elimination by employing artificial aptazymes. Verification of expression control in vivo will further prove the superiority of this technology. This safer REVec technology that can fine-tune expression levels will contribute to establishing gene therapy approaches that are personalized to the patient’s profile.

## Data Availability

All of the data generated or analyzed during this study are included in this published article.
